# Editorial: Biomarkers in Pulmonary Diseases

**DOI:** 10.3389/fmed.2022.790475

**Published:** 2022-03-11

**Authors:** Bassam Mahboub, Rifat Hamoudi, Mahmood Yaseen Hachim, Hauke Busch

**Affiliations:** ^1^Rashid Hospital, Dubai, United Arab Emirates; ^2^College of Medicine, Sharjah Institute for Medical Research, University of Sharjah, Sharjah, United Arab Emirates; ^3^Division of Surgery and Internventional Science, University College London, London, United Kingdom; ^4^Mohammed Bin Rashid University of Medicine and Health Sciences, Dubai, United Arab Emirates; ^5^University of Lübeck, Lübeck, Germany

**Keywords:** artificial intelligence, pulmonary, biomarker, genes, clinical

The incidence of pulmonary diseases such as asthma, chronic obstructive pulmonary disease (COPD), and different types of lung cancer are on the rise worldwide and will most likely continue to do so with ubiquitous air pollution and climate change, including extreme weather conditions. According to the World Health Organization, pulmonary diseases like COPD are among the leading causes of death. Lower respiratory infections are the third and fourth leading causes of death, especially in low-income countries. These diseases present themselves as highly complex and heterogeneous concerning diagnosis, treatment, or even prevention. Therefore, elucidating the disease etiology is essential to identify biomarkers for early detection, treatment, relapse, and a personalized approach to disease management.

This Research Topic gathers different contributions highlighting novel ways of clinical stratification of patients, gathering molecular markers for disease based on molecular diagnostics, novel omics, imaging, and screening technology. These approaches allow us to shed light on clinical outcomes and putative treatment schemes in the future.

Novel molecular markers for asthma, respiratory syndromes, and lung cancer have been proposed through cohort studies. Two case reports suggest novel molecular markers for rare types of lung cancer, which might be ideal proxies for more extensive cohort studies. In five cases of pulmonary sclerosing pneumocytoma, Aramini et al. found elevated levels of ALDH and SOX-2 and provided the first hint at their usefulness as a biomarker. The authors further present two cases of primary angiosarcoma of the lung in which they report on ALDH as a marker for poor outcome as well, which should be confirmed in a larger study.

In a retrospective study of 308 patients, Lu et al. proposed the albumin to globulin ratio as a prognostic factor for non-small cell lung cancer, while Yu et al. correlated PD-L1 expression based on tumor location and TTF-1 expression with poor outcome and potential for immunotherapy.

Molecular markers for respiratory diseases from larger cohort studies were proposed by Zhang R. et al. who present the analysis of a study of 133 pulmonary arterial hypertension (IPAH) patients, in which they found a significantly lower extracellular SOD in patients with BMPR2 mutations and propose this to be a vital antioxidant enzyme in the pathogenesis of IPAH.

Zhang W. et al. confirmed the neutrophil to lymphocyte ratio in a cohort of more than 1100 patients as a predictor for short-term survival in acute respiratory distress syndrome (ARDS).

Using seven clinical variables, Liu et al. developed a survival predictor with online access to evaluate acute respiratory distress syndrome.

Several manuscripts used omics approaches to identify novel markers, as such Kim et al. investigated metabolic fingerprinting for tuberculosis and smoking-induced COPD. In a transcriptome analysis of 60 blood samples from 30 asthmatics vs. healthy controls, Elena-Pérez et al. identified IL5RA as a pharmacogenetic biomarker in asthma. Hachim et al. measured Amphiregulin mRNA and protein in the blood, saliva, and bronchial biopsies samples from asthmatic patients and found its expression to be highly correlated with the disease.

Two papers demonstrate the usefulness of genetic association studies in the detection of novel genes and mechanisms. Raita et al. used genotype data from two large population studies, the INTERVAL and the UK Biobank, comprising more than 500,000 people, and the recent Mendelian randomization approach to confirm a causal effect with a significantly higher risk of asthma with soluble IL6 receptors. Gandhi et al. found a complex genetic association with mutations in surfactant proteins with hypersensitivity pneumonitis in the Mexican population.

With the recent worldwide SARS-CoV-2 pandemic, several papers developed novel diagnostic tools for COVID-19 based on either molecular or imaging data. For example, Chen et al. suggested carcinoembryonic antigen (CEA) in evaluating the severity and prognosis of COVID-19 in a cohort of 46 death and 68 discharged cases from the hospital. Yoo et al. developed a classifier for COVID-19 diagnosis from chest X-ray imaging, while Carvalho et al. derived radiological imaging patterns to quantify the extent of pulmonary involvement in COVID-19.

Imaging has also proven helpful for the diagnosis of the lung and respiratory organs in a clinical context. For this Ye et al. proposed a novel speckle-tracking algorithm for right diaphragm deformation analysis to detect abnormalities in respiratory movement.

Beyond genetic and transcriptomic information, also exosomes, membrane-bound extracellular vehicles (EVs) have moved into the focus of research as putative modulators of respiratory disease. Wang et al. reviewed the potential roles of exosomes in COPD as intercellular communication devices, which will probably gain more attention soon. Zhang R. et al. commented on the role of circulating serum exosomes and their role in inducing pulmonary inflammation during acute lung inflammation.

## Conclusion Thoughts

Over the past 5 years, health sciences and medicine have progressed rapidly and swiftly by the wealth of information coming from understanding mechanisms of diseases.

Most of the advances happened because disease presentations rely on omics, which understand that the genetic makeup of the human being will transform into the transcription of specific messages carried by mRNA to be finally present as protein.

Proteins usually show themselves as a clinical phenotype in a patient with a particular disease presentation.

This wealth of big data was difficult to navigate without using the power of computing sciences and mainly machine learning with artificial intelligence.

When biology meets mathematics, great advances in understanding and progression in problem-solving will happen.

I hope readers will enjoy this unique issue, which touches the surface of what current and future medicine of pulmonary disease looks like.

## Author Contributions

BM, MH, and HB were responsible for the conception of the idea. All authors approved the final manuscript for publication.

## Conflict of Interest

The authors declare that the research was conducted in the absence of any commercial or financial relationships that could be construed as a potential conflict of interest.

## Publisher's Note

All claims expressed in this article are solely those of the authors and do not necessarily represent those of their affiliated organizations, or those of the publisher, the editors and the reviewers. Any product that may be evaluated in this article, or claim that may be made by its manufacturer, is not guaranteed or endorsed by the publisher.

